# Sleep disturbance in Inflammatory Bowel Disease: prevalence and risk factors – A cross-sectional study

**DOI:** 10.1038/s41598-020-57460-6

**Published:** 2020-01-16

**Authors:** C. Marinelli, E. V. Savarino, I. Marsilio, G. Lorenzon, T. Gavaruzzi, R. D’Incà, F. Zingone

**Affiliations:** 1 Department of Surgery, Oncology and Gastroenterology, Gastroenterology Section, University Hospital of Padua, I-35128 Padua, Italy; 2 Department of developmental psychology and socialization, University Hospital of Padua, via Venezia, 8, 35131 Padua, Italy

**Keywords:** Medical research, Inflammatory bowel disease

## Abstract

Inflammatory bowel diseases (IBD) are chronic relapsing disorders that have a negative impact on quality of life. They can be highly disabling and have been associated with sleep disturbance. The aim of our study was to evaluate the sleep quality of a large cohort of IBD patients to identify possible associated cofactors. We prospectively recruited consecutive patients attending the IBD Unit of “Azienda Ospedaliera” of Padua from November 2018 to May 2019 and collected demographics and clinical characteristics. The patients completed the Pittsburgh Sleep Quality Index (PSQI), the IBD questionnaire (IBDQ), the IBD-Disability Index (IBD-DI) questionnaire, and the Hospital Anxiety and Depression Scale (9-HADS). A multivariate regression model was applied to assess independent risk factors of sleep disturbance among IBD-related variables, disability, quality of life, anxiety, and depression. We investigated the sleep quality of 166 patients with IBD, finding 67.5% of them suffering from sleep disturbance. In particular, low quality of life, presence of disability and extraintestinal manifestations were identified as independent risk factors of sleep disturbance. We discovered that all depressed patients were also affected by sleep disturbance, while we found no difference in sleep disturbance between patients with or without anxiety state. However, a positive correlation was reported between both anxiety and depression scores and PSQI score (Spearman correlation: r = 0.31 and r = 0.38 respectively). Our study showed that sleep quality is not directly associated with an active or inactive IBD state or with the ongoing treatment, but it is mostly correlated with the patients’ mood state, disability, and quality of life. Gastroenterologists and psychologists should join forces during clinical outpatients’ visits to evaluate emotional states for a better IBD management.

## Introduction

Inflammatory bowel disease (IBD) is a group of chronic relapsing disorders that includes, principally, Crohn’s disease (CD) and ulcerative colitis (UC) and is characterized by a progressive course and potential development of complications, including extra-intestinal manifestations (EIMs)^1^.

Indeed, both diseases are associated with a marked reduction of health-related quality of life, severe fatigue, and work impairment^2,3^, as well as with depression and anxiety^4^. In particular, changes in mood and mental state are linked to both development and disease worsening in IBD patients^5–10^. Furthermore, sleep, considered as an environmental factor, has been investigated as a possible marker of subclinical inflammation^6,11^. An unsatisfactory sleep quality is commonly reported in the general population, due to several environmental and psychological factors. Alerting factors include older age, female gender, lower socio-economic status, marital status different from being married^12^, anxiety, and lack of optimism^13,14^. Patients with chronic medical disorders report an even worse quality of sleep, experiencing fewer hours and less restorative sleep when compared to healthy individuals^5^. In particular, some evidence suggests that sleep disorder is one of the main concerns of patients suffering from chronic intestinal disease, which can seriously impact health and work productivity in the long term^15,16^. Furthermore, it has been shown that sleep disturbance can worsen the subjective symptoms of the disorder, leading to more frequent relapses^17^.

Thus, a poor sleep quality is associated with troubles and distress in patients with IBD^18^, resulting in an impaired quality of life^19^. However, only a few studies^6,20,21^ have analyzed the risk factors associated with sleep disturbance in IBD patients. The aim of the present study was to evaluate sleep disturbance in IBD patients and identify its risk factors, including disease-related variables and mood states.

## Results

### Demographics and clinical characteristics

A total of 166 subjects completed the Inflammatory Bowel Disease Questionnaire (IBDQ) for quality of life assessment, a global score ≤170 is expression of low QoL; the IBD-Disability Index (IBD-DI) questionnaires for disability assessment, and a global score ≤3.5 is expression of greater disability, and the Pittsburgh Sleep Quality Index (PSQI) for sleep quality assessment. In particular, the latter is a self-reported survey that evaluates characteristics such as sleep duration, efficiency, and disturbances. A global score ≥5 reveals poor sleep quality.

Table 1 shows demographics and baseline characteristics of our population. The mean age was 44.39 years (±13.92; range 18–80). In particular, 82 patients (49.40%) were aged more than 45 years. Sex distribution was 79 females (47.59%) and 87 males (52.41%). In 134 patients (80.7%) disease was considered clinically in remission, although 47% of all population had calprotectin levels higher than 250. Forty-five (27.11%) patients were affected by at least one EIM associated to IBD with only 16 of them having an active disease at the time of the study.

**Table 1:** Socio-demographic and clinical characteristics of population.

	N = 166 (%)
**Variables:**	
Disease	
Crohn’s Disease	87 (52.1)
Ulcerative colitis	79 (47.59)
Age group (≥45)	82 (49.4)
Sex (Men)	87 (52.41)
Current smoking status	
Non-smoker/ex-smoker	142 (85.54)
Smoker	24 (14.46)
Occasional alcohol intake (Yes)	88 (53.01)
Active Disease	
(pMayo >1 or HBI >4)	32 (19.28)
High Faecal Calprotectin (>250)	79 (47.6)
Anaemia	
(Hb <12 females, <13 males)	23 (13.86)
UC localization	
E1/E2	35 (44.1)
E3	44 (55.44)
CD Behavior	
Nonstricturing, nonpenetrating	36 (41.04)
Stricturing	38 (43.32)
Penetrating	13 (14.82)
Localization	
L1 terminal ileum	24 (27.36)
L2 colon or L3 ileocolon	59 (67.24)
L4 Upper or upper + other	4 (4.56)
Presence of extra-intestinal manifestations	45 (27.11)
Immunosuppressant: on-going use	29 (17.47)
Biologics: on-going use	98 (59.04)
Abdominal Surgery (Yes)	48 (28.92)
IBDQ pathological (≤170)	74 (44.58)
IBD-DI pathological (≤3.5)	90 (54.22)
PSQI pathological (≥5)	112 (67.5)

Abbreviations: IBD questionnaire (IBDQ), IBD-Disability Index (IBD-DI), Pittsburgh Sleep Quality Index (PSQI).

At the time of the study, 98 (59.4%) patients were receiving therapy with biologic drugs (Infliximab, Adalimumab, Vedolizumab or Golimumab) and 29 (17.47%) with immunosuppressants (Azathioprine/AZA or Metotrexate). Subjects with a low IBDQ score (≤170) were 74 (44.58%); subjects with a low IBD-DI score (≤3.5) were 90 (54.22%) (Table 1).

### Sleep, quality of life, and disability assessment

Patients who reported a pathological score of PSQI (≥5) were 112 (67.5%) (Table 1). In particular, we found that 64.9% (87/134) of patients in clinical remission and 78.1% (25/32) of patients with active disease (p = 0.15) had poor sleep quality. Considering each sleep component of PSQI, we found the highest sub-score in sleep disturbances (mean 1.49, SD 0.6) and the lowest one in habitual use of sleep medication (mean 0.33, SD 0.9) (Table 2). Sixty-two (55.4%) patients with bad sleep quality also had a poor quality of life, whereas 65.2% (73/112) had a low disability score. Considering each sub-score of both IBDQ and IBD-DI questionnaires, patients with poor sleep quality had statistically significant lower scores compared to patients reporting no sleep disturbance except for one IBD-DI sub-score: body structures (Supplementary Material, Tables S1 and S2).

**Table 2:** PSQI results.

	Median (25–75^th^ percentile)	Mean (SD)
global score	6 (0–17)	6.45 (3.68)
subjective sleep quality	1 (4–9)	1.21 (0.7)
sleep latency	1 (0–2)	1.09 (0.9)
sleep duration	1 (0–2)	1.03 (0.9)
habitual sleep efficiency	0 (0–1)	0.60 (0.9)
sleep disturbances	1 (1–2)	1.49 (0.6)
use of sleeping medication	0 (0–0)	0.33 (0.9)
daytime dysfunction	0 (0–1)	0.80 (0.7)

Table 3 describes the factors that were significantly associated with the PSQI score. Based on the univariate analysis, we observed that patients who suffered from extra-intestinal manifestations, patients with pathologic IBD-DI score (≤3.5), with lower IBDQ score (≤170), with no occasional alcohol consumption and females were more likely to have a PSQI score ≥5 (Table 3). Age, type of disease (CD or UC), disease extent and phenotype, ongoing treatment, low hemoglobin levels, and elevated faecal calprotectin were instead not identified as risk factors for bad sleep quality (data not shown). We designed two different multivariate regression models including IBDQ and IBD-DI groups separately, together with the other significant risk factors found in the univariate analysis (presence of EIMs, alcohol consumption and sex), to respect the rules of multi-collinearity (Spearman correlation between IBD-DI and IBDQ scores were >0.80). Using the two models, only IBDQ, IBD-DI and EIMs were identified as independent risk factors of sleep disturbance. In particular subjects with low scores of IBD-DI and IBDQ were between three and four times more likely to have poor sleep quality (OR 3.6 95% CI 1.77–7.29 and OR 3.8, 95% CI 1.78–8.10, respectively). Moreover, in both models, patients with EIMs had a risk of sleep disturbance more than twice higher compared to patients without EIMs (Table 3).

**Table 3:** Variables resulted statistically significant associated with PSQI in all population.

Variables:	Univariate analysis	Multivariate model with IBDQ	Multivariate model with IBD-DI
	Unadjusted OR *Risk of having pathological PSQI* (≥*5*)	Adjusted OR *Risk of having pathological PSQI* (≥*5*)	Adjusted OR *Risk of having pathological PSQI* (≥*5*)
Sex			
Men	1	—	—
Women	2.12 (1.08–4.15)		
Occasional alcohol intake			
No	1	—	—
Yes	0.48 (0.25–0.95)		
Extra-intestinal manifestations			
No	1	1	1
Yes	3.44 (1.42–8.35)	2.73 (1.08–6.83)	2.75 (1.09–6.88)
IBDQ			
>170	1	1	/
≤170	4.34 (2.06–9.11)	3.80 (1.78–8.10)	
IBD-DI			
>3.5	1	/	1
≤3.5	4.07 (2.03–8.15)		3.59 (1.77–7.29)

Abbreviations: IBD questionnaire (IBDQ), IBD-Disability Index (IBD-DI).

**Figure 1:**
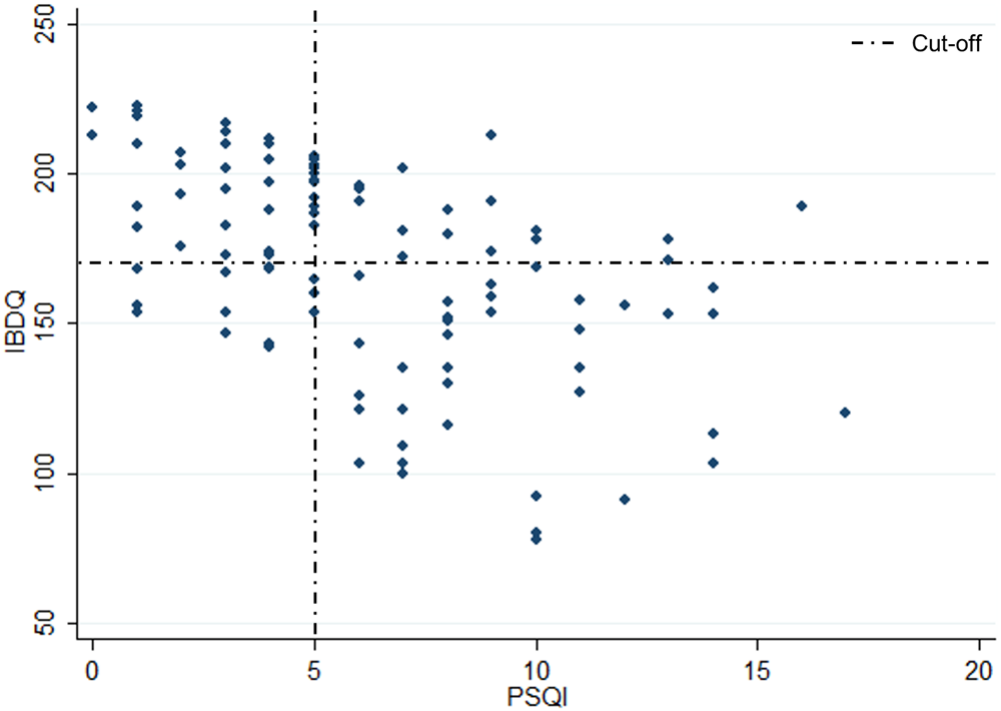
Spearman correlation between IBDQ and PSQI scores.

**Figure 2:**
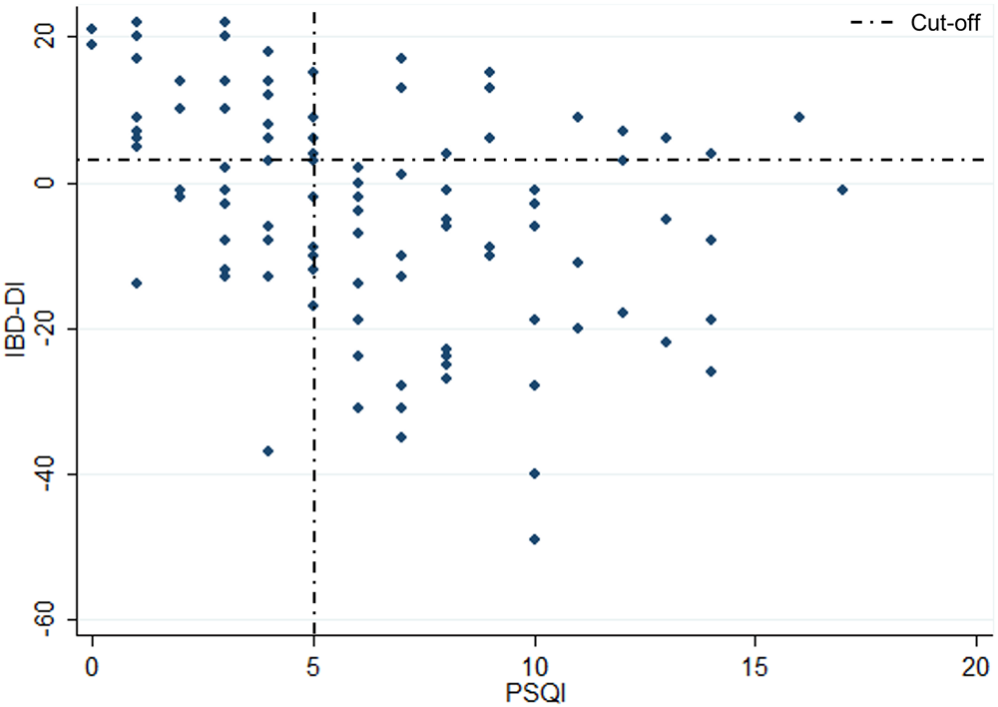
Spearman correlation between IBD-DI and PSQI scores.

As shown in Table 4, we observed a moderately strong negative correlation between PSQI score and IBDQ (r −0.50, p < 0.001) (Fig. 1), and between PSQI and IBD-DI (r −0.45, p < 0.001) (Fig. 2).

**Table 4:** Spearman’s rank correlations coefficient (95% CI).

	Age	pMayo	HBI	Faecal Calprotectin	Hb	IBDQ score	IBD-DI score
PSQI score	0.17*	0.14	0.07	−0.14	−0.16	−0.50**	−0.45**

**p < 0.01; *p < 0.05.

Abbreviations: Pittsburgh Sleep Quality Index (PSQI).

### Sleep and mood state (depression and anxiety) assessment

A sub-group of patients (n = 74: 35 females, mean age 44.3 ± 13.23, 35 UC) also completed the HADS scale. Among these, 31 patients showed anxiety and 8 depressive mood. We found that all patients with depressive mood had sleep disturbance (100%) while it was present in 66.7% of those without depression (p = 0.05). In contrast, we did not find any statistically significant difference in sleep disturbance between patients with and without anxiety (p = 0.25) (Table 5). However, we observed a moderately positive correlation between PSQI and HADS score for both anxiety (r 0.31, p 0.006) and depression (r 0.38, p < 0.001) in the subset of patients.

**Table 5:** Quality of sleep in a subset of 74 patients with HADS assessment.

	n = 74	Sleep disturbance (PSQI score ≥5)	P
Depression			
No (score <8)	66	44 (66.7%)	0.05
Yes (score ≥8)	8	8 (100%)	
Anxiety			
No (score <8)	43	28 (65.1%)	0.25
Yes (score ≥8)	31	24 (77.4%)	

## Discussion

### Main results

While it has been long recognized that IBD patients suffer from poor sleep quality, only a handful of studies^6,20,21^ have evaluated the prevalence and risk factors associated with sleep disturbance and severity in inflammatory bowel condition^4,15^. For this reason, we decided to investigate the sleep quality of 166 patients with IBD using validated questionnaires. We found that two thirds of patients with IBD suffered from sleep disturbance. Poor sleep quality was associated with low quality of life, greater disability and presence of EIMs whereas it did not appear to be associated to disease variables such as subtype, disease activity, or type of therapy taken. Absence of correlation with IBD-related characteristic can be due to a generalized state of discomfort. Furthermore, we showed that all our depressed patients were affected by sleep disturbance, while an anxiety state did not significantly impact on sleep quality. However, a positive correlation was found between PSQI score and both anxiety and depression scores.

### Previous literature

We found a prevalence of sleep disturbances in IBD of 67.5%, which is higher than that described in other studies on the healthy population^22^. We did not find an association between the two major forms of IBD and PSQI pathologic score. This is in line with previous evidence that reports no difference in sleep quality disturbance between CD and UC patients (6.8 ± 3.4 versus 7.4 ± 3.1; p = 0.48)^20^. Conversely, we did not confirm the previous evidence that associates being female with more frequent PSQI pathologic score^20^. We only found a difference in sleep quality in the univariate analysis and it was not confirmed in the multivariate ones. This difference in sleep quality might be due to the gender differences in the prevalence of psychiatric morbidities, socio-cultural factors, and coping strategies^23^. We found that all subjects with depression had sleep disturbance and that a moderate positive correlation between anxiety and depression scores and PSQI score was present. These results are in line to those described in the general population^24–26^. For this reason, psychological factors can be associated with a bad sleep quality in IBD patients, more than with the disease *per se*.

Notably, IBD patients could suffer from sleep problems linked to medication side effects. For this reason, several studies regarding sleep and fatigue have previously taken into consideration the ongoing therapy. In 2018, Lee *et al*. assessed sleep disturbance in IBD patients receiving immunosuppressant and biologic drugs^27^. None of these therapies turned out to be predictive of disturbed sleep. This is in line with our findings highlighting that sleep quality is not associated with the current treatment. Sobolewska *et al*. investigated sleep quality in a small cohort of IBD patients. They found that poor sleep was assessed in 78% of clinically-active patients (40/51) and in 35% of patients considered in remission (5/14) (p = 0.002; OR 6.5, 95% CI 1.8–23.6)^20^. Conversely and analogous to our findings, Ranjbaran *et al*. used survey data to demonstrate that poor sleep quality and poor quality of life are associated also in patients which are clinically considered in remission^21^. Similar results were also found by Bar-Gil Shitrit *et al*.^28^. Furthermore, a previous study reported that poor sleep was more common in IBD patients with high C-Reactive Protein levels than in those with normal values^29^, while we did not find an association between quality of sleep and faecal calprotectin. However, only this latter is considered a specific inflammation marker for IBD^30^. Therefore, we did not find an association either with clinical or with biochemical signs of disease activity. Finally, certain EIMs, such as peripheral arthritis or erythema nodosum, are frequently associated with active intestinal inflammation while other EIMs, such as uveitis or ankylosing spondylitis, usually occur independently of intestinal inflammatory activity^31^. We found an independent association between sleep disturbance and presence of EIMs but not between sleep disturbance and active disease. This result can be justified by the fact that in our population only 35 patients with EIM had an active disease at the time of the study. We have recently published a study which reports the presence of EIMs as an independent risk factor of poor QOL and presence of disability^32^.

### Strengths and limitations

Our study has multiple strengths. First, it is based on a large population referring to the same IBD Unit, thus different environmental factors as well as different diseases approaches did not influence our results. Moreover, we applied state-of-the-art methods to assess all the variables investigated, while the questionnaires were all administered by one interviewer in order to ensure that all patients understand the questions. Finally, to the best of our knowledge, this is the first study aimed at assessing sleep disturbance associated with disability and mood disorders in IBD patients.

We have also identified a number of limitations. First, the results of our investigations are based on self-reported measures and lack an objective measurement of sleep (i.e. polysomnography). However, it is relevant to note that, in the past, Keefer *et al*. have demonstrated a good correlation between polysomnography measure and subjective sleep perception investigated with PSQI questionnaire^21,33^. Second, our results were not associated with objective evidence of disease (endoscopy), however we used faecal calprotectin level which has been considered a good alternative to endoscopy evaluation^34^. Third, a cross-sectional protocol provides limited evidence concerning changes over time, which would be better assessed by a future longitudinal study. Moreover, considering that IBD subjects often have overlapping IBS^35^, another limitation is the lack of assessment of IBS in our study population. Finally, we only started administering the HADS questionnaire after the study had started, meaning that it was only completed by a subset of patients. However, in the absence of a selection bias (all consecutive patients have also completed this questionnaire since February 2019), we believe that this sub-group is still representative of the entire population.

## Conclusions

We demonstrated that a poor quality of life in IBD patients is strongly related to poor quality of life, disability, and mood state. In IBD management not only disease progression, but also sleep and disability should be investigated during clinical outpatients’ visits. This would also allow for a novel multidisciplinary approach based on collaborative efforts between gastroenterologists and psychologists with the aim to improve sleep and quality of life in general, and to reduce disability and the restriction and limitations that sleep deprivation can induce in daily life.

## Methods

### Patients

The present cross-sectional study was approved by the Ethics Committee of Padua (protocol number 4197/AO/17) in November 2018 and procedures applied were in conformity with a previous study we conducted^32^. We consecutively and prospectively recruited all IBD patients who visited the IBD Unit of the “Azienda Ospedaliera” of Padua from November 2018 to May 2019. Inclusion criteria were age ≥18 years and a confirmed diagnosis of UC or CD based on clinical, endoscopic and histological examinations according to international criteria^36^ from at least six months. The exclusion criteria were patients diagnosed with cognitive disorders or psychiatric illness, stomas, history of alcoholism, pregnancy, and patients who refused to sign the informed consent form.

All participants were informed about the nature, duration, and purpose of the study. The study protocol was performed accordingly to the ethical guidelines of the 1964 Declaration of Helsinki (6^th^ revision, 2008) as reflected in a priori approval by the institution’s human research committee.

All patients enrolled were asked to complete the validated Italian versions of the Pittsburgh Sleep Quality Index (PSQI)^37^ and of the Inflammatory Bowel Disease Questionnaire (IBDQ)^38^ to respectively evaluate sleep disorder and quality of life. The IBD-Disability Index (IBD-DI) questionnaire, used as tool to assess disability, was administered by an interviewer since the questionnaire was not validated in Italian language^1^and was not designed as a self-reported questionnaire^39^. Furthermore, since February 2019 the patients have also been asked to complete the Hospital Anxiety and Depression Scale (HADS) to determine anxiety and depression. All questionnaires were dispensed with the support of one interviewer (CM) in order to ensure that all patients understand the questions.

### Clinical IBD assessment

Outpatient medical records were used as source of demographics and clinical data (including duration, location phenotypes, and extension of intestinal disease according to Montreal classification^40^), while other information were taken in collaboration with the participants. For UC patients, the partial Mayo score was used to assess disease activity, while the Harvey Bradshaw Index was used for CD patients. Disease activity was also determined through faecal calprotectin measurement, as non-invasive marker^34,41^. Supplementary clinical data, like age at symptoms’ onset and at diagnosis, extra-intestinal manifestations associated to the IBD, history of IBD-related surgery, and current medical therapy were also collected.

### Sleep disorders assessment

Patients were asked to complete the Pittsburgh Sleep Quality Index (PSQI) in Italian language to evaluate sleep quality and related disturbances^37^, considering events occurred over the previous month. The PSQI consists of 7 “component” scores, including subjective sleep quality, use of sleeping drug, and daytime disorders. Patients can answer “0” in case of no difficulty or “3” in case of severe difficulty for each component score. The sum of each component score produces a total score that ranges from 0 to 21. When it results ≥5, it is suggestive of significant bad sleep quality^42^.

### Quality of life assessment

Patients completed the validated Italian version of the Inflammatory Bowel Disease Questionnaire (IBDQ) for general quality of life measurement^38^, often used as tool for treatment outcome measurement^2,43–45^. The IBDQ explores not only intestinal, but also emotional function (such as depression, anxiety, anger, and embarrassment), systemic symptoms (such as sleep disorders and fatigue) and social function. The sum of each component produces a total score representing poor or good quality of life (range 32–224). Scores higher than 170 were previously associated with patients with clinically inactive disease^2^.

### Disability assessment

Patients fulfilled the IBD disability index (IBD-DI) that consists of 19 items organized in 28 parts. Health and disability sections are overall health, body functions (sleep/energy/fatigue, affect, body image, pain, diarrhea, body mass index, weight loss), activities and participation (regulating defecation, looking after one’s health, interpersonal activities, and work/education), body structures (blood in stool, arthralgia/arthritis), and environmental factors (exacerbating effect of medication, food, family, and professional health care)^39^.

The total score on the IBD disability represents maximum state of disability index or no disability (range −80–22). A previous study identified a cut-off of 3.5 as the differentiation point for IBD versus healthy controls^1^.

### Anxiety and depression

Anxiety and depression were assessed using the Hospital Anxiety and Depression Scale (9- HADS) questionnaire first used by Zigmond and Snaith^46^ and validated in Italian in 1999^47^. This instrument is a self-report questionnaire composed of 7 items to assess depression and 7 items for anxiety within the previous week. Each item score ranges from 0 to 3. The sum of each component score produces a total score for depression and a total score for anxiety (range 0–21). Higher global scores indicate worst symptoms. A score of ≥11 is considered a clinically significant disorder, whereas a score between 8 and 10 suggests a mild disorder^46^. In our population, we considered patients with a score ≥8 as having an anxiety and depression mood.

### Statistical analysis

Categorical variables were expressed as frequency and continuous variables as mean ± Standard Deviation (SD) and median with 25–75^th^ percentiles. Differences in categorical variables were calculated using Chi Square Test. We used univariate logistic regression models to assess whether demographical, IBD related variables, IBDQ, and IBD-DI scores were related to a pathological PSQI (score ≥5). Statistically significant variables in the univariate analyses were then included in a multivariate regression model to identify, using the backward elimination analysis, the independent risk factors of sleep disturbance. We used the Spearman’s rank correlations coefficient to evaluate the correlations between PSQI and the following variables: age, partial Mayo score, HBI, faecal calprotectin, hemoglobin, IBD-Q score, IBD-DI score, anxiety, and depression. A p value < 0.05 was considered statistically significant. STATA 11 software was used to perform statistical analysis.

### Ethical approval

All procedures performed in studies involving human participants were in accordance with the ethical standards of the institutional and/or national research committee and with the 1964 Helsinki declaration and its later amendments or comparable ethical standards.

### Informed consent

Informed consent was obtained from all individual participants included in the study.

## Supplementary information

Table S1.

Table S2.

## Data Availability

The datasets used and/or analyzed during the current study are available from the corresponding author on reasonable request.
