# Supplementary material for: Sleep disturbance in Inflammatory Bowel Disease: prevalence and risk factors – A cross-sectional study
Source: Sci Rep. 2020 Jan 16;10:507. doi: 10.1038/s41598-020-57460-6 (PMC6965196; doi:10.1038/s41598-020-57460-6)
Supplement: Supplementary file 1 — Table S1. [file 41598_2020_57460_MOESM1_ESM.docx]

**Sleep disturbance in Inflammatory Bowel Disease: prevalence and risk factors – A cross-sectional study**

**Authors**: Marinelli C., Savarino E.*, Marsilio I., Lorenzon G., Gavaruzzi T., D’Incà R., Zingone F.

**Table S1 IBDQ sub-scores associated with PSQI**

| IBDQ sub-score | PSQI < 5 group (mean±SD) | PSQI ≥5 group (mean±SD) | P |
| --- | --- | --- | --- |
| Intestinal function | 61.5 ± 7.6 | 54.7 ± 10.6 | <0.001 |
| Systemic function | 27.3 ± 5.3 | 21.9 ± 6.3 | <0.001 |
| Emotional function | 69.4 ± 10.2 | 57.7 ± 1.6 | <0.001 |
| Social function | 32.5 ± 3.8 | 28.7 ± 6.9 | <0.001 |
