# Supplementary material for: Sleep disturbance in Inflammatory Bowel Disease: prevalence and risk factors – A cross-sectional study
Source: Sci Rep. 2020 Jan 16;10:507. doi: 10.1038/s41598-020-57460-6 (PMC6965196; doi:10.1038/s41598-020-57460-6)
Supplement: Supplementary file 2 — Table S2. [file 41598_2020_57460_MOESM2_ESM.docx]

**Sleep disturbance in Inflammatory Bowel Disease: prevalence and risk factors – A cross-sectional study**

**Authors**: Marinelli C., Savarino E.*, Marsilio I., Lorenzon G., Gavaruzzi T., D’Incà R., Zingone F.

**Table S2. IBD-DI sub-scores associated with PSQI**

| IBD-DI sub-score | PSQI < 5 group (mean±SD) | PSQI ≥5 group (mean±SD) | P |
| --- | --- | --- | --- |
| Overall health | -1.29 ± 0.8 | -1.7 ± 0.8 | <0.001 |
| Body function | -3.5 ± 4.1 | -9.2 ± 5.8 | <0.001 |
| Activities and partecipation | -2.1 ± 2.8 | -4.7 ± 4.0 | <0.001 |
| Body structures | 1.07 ± 1.3 | 0.8 ± 1.2 | 0.17 |
| Environmental factors | 12.6 ± 6.2 | 19.2 ± 6.4 | 0.02 |
